# Analysis of the Neurotoxin Complex Genes in *Clostridium botulinum* A1-A4 and B1 Strains: BoNT/A3, /Ba4 and /B1 Clusters Are Located within Plasmids

**DOI:** 10.1371/journal.pone.0001271

**Published:** 2007-12-05

**Authors:** Theresa J. Smith, Karen K. Hill, Brian T. Foley, John C. Detter, A. Christine Munk, David C. Bruce, Norman A. Doggett, Leonard A. Smith, James D. Marks, Gary Xie, Thomas S. Brettin

**Affiliations:** 1 Integrated Toxicology Division, United States Army Medical Institute of Infectious Diseases, Fort Detrick, Maryland, United States of America; 2 Bioscience Division, Los Alamos National Laboratory, Los Alamos, New Mexico, United States of America; 3 Theoretical Division, Los Alamos National Laboratory, Los Alamos, New Mexico, United States of America; 4 Department of Anesthesia and Pharmaceutical Chemistry, University of California at San Francisco, San Francisco, California, United States of America; University of Maryland, United States of America

## Abstract

**Background:**

*Clostridium botulinum* and related clostridial species express extremely potent neurotoxins known as botulinum neurotoxins (BoNTs) that cause long-lasting, potentially fatal intoxications in humans and other mammals. The amino acid variation within the BoNT is used to categorize the species into seven immunologically distinct BoNT serotypes (A–G) which are further divided into subtypes. The BoNTs are located within two generally conserved gene arrangements known as botulinum progenitor complexes which encode toxin-associated proteins involved in toxin stability and expression.

**Methodology/Principal Findings:**

Because serotype A and B strains are responsible for the vast majority of human botulism cases worldwide, the location, arrangement and sequences of genes from eight different toxin complexes representing four different BoNT/A subtypes (BoNT/A1-Ba4) and one BoNT/B1 strain were examined. The bivalent Ba4 strain contained both the BoNT/A4 and BoNT/bvB toxin clusters. The arrangements of the BoNT/A3 and BoNT/A4 subtypes differed from the BoNT/A1 strains and were similar to those of BoNT/A2. However, unlike the BoNT/A2 subtype, the toxin complex genes of BoNT/A3 and BoNT/A4 were found within large plasmids and not within the chromosome. In the Ba4 strain, both BoNT toxin clusters (A4 and bivalent B) were located within the same 270 kb plasmid, separated by 97 kb. Complete genomic sequencing of the BoNT/B1 strain also revealed that its toxin complex genes were located within a 149 kb plasmid and the BoNT/A3 complex is within a 267 kb plasmid.

**Conclusions/Significance:**

Despite their size differences and the BoNT genes they contain, the three plasmids containing these toxin cluster genes share significant sequence identity. The presence of partial insertion sequence (IS) elements, evidence of recombination/gene duplication events, and the discovery of the BoNT/A3, BoNT/Ba4 and BoNT/B1 toxin complex genes within plasmids illustrate the different mechanisms by which these genes move among diverse genetic backgrounds of *C. botulinum*.

## Introduction

*Clostridium botulinum* is a taxonomic collection of several distinct species of anaerobic gram positive spore-forming bacteria which produce the most poisonous naturally occurring substance known, botulinum neurotoxin (BoNT) [1]. The organisms have been divided into four distinct genetic and physiological groupings [2] and 16s rDNA sequencing has shown these groupings to be separate species [3], [4]. The neurotoxins produced by these organisms are categorized serologically into seven distinct groups designated serotype A through G [2]. Four of the seven serotypes, A, B, E, and F, cause human botulism with the vast majority of cases due to serotypes A and B [5].

The neurotoxins cause the disease botulism, a rare but potentially fatal intoxication that occurs in three forms: foodborne botulism, infant botulism, and wound botulism. Intoxications are long-lived, persisting from several weeks to several months, and victims may require intensive care, including mechanical ventilation, for extended periods of time [6]. Because of their extreme toxicity and prolonged effects, botulinum neurotoxins are grouped as CDC category A select agents [7]. In contrast, this extremely toxic protein has been employed to treat a wide variety of muscle and nerve disorders, including arthritis and migraine headaches [8].

*C. botulinum* strains, and the neurotoxins they produce, are highly diverse. Subtypes have been identified within six of the seven serotypes based on amino acid variation within the BoNT. While serotypes differ by about 35–70%, subtype differences range from approximately 2–32%. This diversity can account for significant differences in protection with vaccines and therapeutic agents [9], [10]. Although less common, bivalent strains that express two different BoNT types exist and are designated by the predominant toxin produced (Ab, Ba, Af, Bf) [11]. Other bivalent strain variants such as the A1(B) strains contain both BoNT/A and BoNT/B genes but express only BoNT/A [12]. In addition to the variation within *C. botulinum*, BoNTs found in other species such as *C. baratii* (BoNT/F) and *C. butyricum* (BoNT/E) have resulted in disease[5].

The BoNT is associated with several proteins in arrangements known as botulinum progenitor neurotoxin gene clusters. The botulinum progenitor neurotoxin gene clusters are approximately 11–14 kb and encode for 3–7 proteins that form the neurotoxin complex. All toxin clusters contain a nontoxin-nonhemagglutinin (NTNH) protein that is closely associated with the BoNT protein. Some toxin clusters have associated HA proteins with hemagglutinating properties, while others lack these proteins. One of the proteins, BotR, is known to be a regulatory protein that controls the expression of several of the other toxin cluster proteins [13]. The functions of the associated nontoxic proteins are not fully understood but it is thought that these proteins assist in stability of the neurotoxin within the acidic and protease-rich environment of the stomach and assist in transport of the toxin from the intestinal area to the bloodstream [14].

The arrangement and sequence of these toxin complex genes in relation to the BoNT gene has been described for all seven serotypes and for several subtypes within these serotypes. Serotype A subtypes exhibit two different toxin complex gene arrangements. The BoNT/A1 subtype contains polycistronic hemagglutinins HA70, HA17, HA33, and BotR, NTNH and BoNT genes [15]. This same arrangement is also found in strains of serotype B [16], [17] and is similar to the arrangement within serotype G [18] and C and D strains [19], [20]. In contrast, the BoNT/A2 subtype toxin gene cluster contains polycistronic open reading frames orfX3, orfX2, orfX1, and BotR, p47, NTNH, and BoNT genes [21]. This arrangement has also been described in serotype F strains [17], [22] and the A1 neurotoxin cluster from A1(B) strains [15]. A similar arrangement, but lacking the BotR gene, is found within *C. botulinum* type E and BoNT/E-producing *C. butyricum* strains [23].

Although the overall arrangement of the BoNT complex genes within the different serotypes is similar, the DNA and amino acid sequences within these genes can differ greatly from each other. Differences among serotypes [24], among subtypes within a serotype [23], and within a subtype [25] have all been described. For example, the toxin complex genes of BoNT/A1 and BoNT/C both contain HA70, HA17, HA33, BotR, NTNH and BoNT. However, comparisons of the amino acid identities for these proteins show they range from only 29%–67% [15], [19]. In contrast, the amino acid identities for the toxin cluster within BoNT/A1 and BoNT/B1 subtypes are quite high (82%–98%) for all non-toxin cluster proteins but surprisingly low (37%) for the toxin [15], [26]. The BoNT proteins are particularly divergent, with amino acid homologies for the BoNT serotypes ranging from 34–64% [19]. In contrast, NTNH amino acid homologies from all seven serotypes range from 58%–100%, illustrating the conserved nature of this protein [18].

Comparisons among the genes within the toxin cluster provide an insight into the relationships of the different serotypes and identify regions of genetic recombination and sequence conservation. To further understand the relationships of the toxin cluster genes in serotype A and B strains, whole genome sequencing of several *C. botulinum* strains were undertaken. Sequences representing the four different BoNT/A subtypes (BoNT/A1-A4), including a bivalent Ba4 strain that produces both bivalent BoNT/B and BoNT/A4, and one BoNT/B1 strain were analyzed and compared with the published sequence from BoNT/A1 strain ATCC 3502 (Hall 174) [4]. The similarity of these BoNT/A1-A4 and BoNT/B1 complex genes to each other, their intergenic regions and flanking regions, and their location within the genome or within a plasmid is presented.

## Materials and Methods

### Strains

A total of six strains representing the four different subtypes of BoNT/A (BoNT/A1-A4), and a BoNT/B1 were selected for genomic sequencing. These included two BoNT/A1 strains (Hall and ATCC 19397), the BoNT/A2 Kyoto-F strain, the BoNT/A3 Loch Maree strain, a bivalent Ba4 strain (strain 657) and a BoNT/B1-producing strain (okra). The histories of the two BoNT/A1 strains are unknown but most likely originated from foodborne botulism cases in the western US. The Kyoto-F strain was associated with an infant botulism case in Japan [27]. The Loch Maree strain was isolated from a food poisoning incident in Scotland [28]. The bivalent Ba4 strain (657) was from an infant botulism case in Texas [29]. The BoNT/B1 okra strain was originally obtained from the National Institute of Health [26] and is presumed to be from a foodborne botulism incident in the US. Additional details about these strains have been published previously [30].

### DNA cloning

DNA from each strain was isolated as previously described [30]. Isolated DNA was randomly sheared to 2–3 or 6–8 kb fragments using a HydroShear™ (Genomic Solutions, Ann Arbor, MI). The sheared DNA was blunt-end repaired, separated on an agarose gel, and extracted and purified using QIAquick™ Gel Extraction Kit (Qiagen, Valencia, CA). Purified fragments were ligated into the *Sma* I site of pUC 18 (Roche, Pleasanton, CA) and electroporated into DH10B Electromax™ cells (Invitrogen Corp., Carlsbad, CA). Transformed cells were selected from LB agar plates containing 100 µg/ml of ampicillin, 120 µg/ml of IPTG, and 50 µg/ml of X-GAL. Recombinant colonies were used to inoculate 384-well microtiter plates for plasmid amplification.

*E. coli* cultures were amplified by rolling circle amplification (RCA) using the Templiphi™ DNA Sequencing Template Amplification Kit (GE Healthcare, Piscataway, NJ). The amplified plasmids were sequenced with standard M13 −28 or −40 primers using the BigDye sequencing kit (Applied Biosystems Inc., Foster City, CA). The reactions were purified by a magnetic bead protocol (see research protocols, <http://www.jgi.doe.gov) and run on an ABI PRISM 3730xl capillary DNA sequencer (Applied Biosystems Inc. Foster City, CA).

Sequences were assembled with parallel phrap (High Performance Software, LLC). Possible mis-assemblies were corrected with Dupfinisher [31] or by analysis of transposon insertions in bridge clones. Gaps between contigs were closed by editing, custom primer walk or PCR amplification. Multiple sequence alignments for phylogenetic tree analyses and recombination analyses were constructed using MUSCLE (http://www.drive5.com/muscle/) [32], with hand editing of difficult regions using BioEdit (http://www.mbio.ncsu.edu/BioEdit/bioedit.html). Similarity Plots were generated using SimPlot software (http://sray.med.som.jhmi.edu/SCRoftware/simplot/) [33].

### Genome annotation

Annotation of the assembled genome sequence was carried out with the genome annotation system GenDB [34]. A combined gene prediction strategy was applied by means of the GLIMMER 2.0 system and the CRITICA program suite [35] along with postprocessing by the RBSfinder tool [36]. tRNA genes were identified with tRNAscan-SE [37]. The deduced proteins were functionally characterized by automated searches in public databases, including SWISS-PROT and TrEMBL [38], Pfam [39], TIGRFAM [40], InterPro [41], and KEGG [42]. Additionally, SignalP [43], helix-turn-helix [44], and TMHMM [45] were applied. Finally, each gene was functionally classified by assigning clusters of orthologous groups (COG) number and corresponding COG category [46] and gene ontology numbers [47].

### Genomic comparison

The whole genome sequences of the *C. botulinum* type A1 strains were deposited within the NCBI website and are available using accession numbers CP000726 (ATCC 19397) and CP000727 (Hall), and the sequence of ATCC 3502 was accessed at www.sanger.ac.uk/Projects/C_botulinum/. Homology searches were conducted at the nucleotide and amino acid sequence level using BLAST [48].

To obtain a list of orthologs from *C. botulinum* genomes, a perl script that determines bidirectional best hits was written. For example, genes g and h are considered orthologs if h is the best BLASTP hit for g and vice versa. E values of ≤10^−15^ were acceptable. A gene is considered strain specific if it has no hits with an E value of 10^−5^ or less. The genome comparisons at the nucleotide level were carried out with genome alignment tools, including MUMmer2 [49], NUCmer [50], and the Artemis Comparison Tool (ACT) [51] (http://www.sanger.ac.uk/Software/ACT/). Insertion sequence (IS) elements were identified and classified by using the ISFinder database [52].

## Results

DNA preparations of two BoNT/A1 strains (Hall and ATCC 19397), the BoNT/A2 Kyoto-F strain, the BoNT/A3 Loch Maree strain, the bivalent Ba4 657 strain, and the BoNT/B1 okra strain were cloned and sequenced to determine the arrangement and sequence variation within the toxin cluster genes and their flanking regions. These strains represent the four known BoNT subtypes within serotype A, plus the bivalent B (bvB) and B1 subtypes. The sequence data analyzed consisted of full-length genomic sequences (1 contig; 3.6–4.0 mb) for four strains (two BoNT/A1, BoNT/A3 and BoNT/B1 strains), 3 contigs (BoNT/Ba4 strain), or 16 contigs (BoNT/A2 strain). The presence of small (10.5 kb) or large (149–270 kb) plasmids were identified within some of the strains. These plasmid contigs were determined to be circular due to the presence of multiple clones containing sequence data from both the beginning and end of the contig within the same clone. The toxin cluster within the BoNT/A2 strain was identified in the middle of a 985 kb contig. Because of the large size of this contig it is assumed that the A2 toxin cluster resides within the chromosome of this strain, similar to the A1 strains.

### Gene Cluster Arrangements

**Figure 1:**
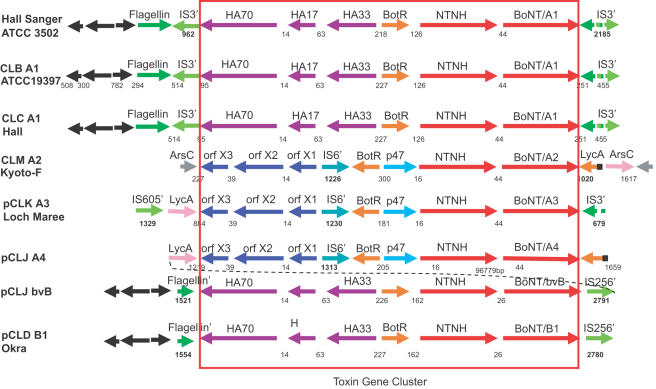
Diagram illustrating the neurotoxin complex gene arrangements found within strains representing the four different BoNT/A1-A4 and the BoNT/B1 subtypes of *C. botulinum.* The gene arrangements within the three BoNT/A1 strains are identical to each other and to the BoNT/B1 okra strain which includes the HA70, HA17, HA33, BotR, NTNH and BoNT genes. This arrangement is different from the three strains that represent the BoNT/A2, BoNT/A3 and BoNT/A4 subtypes which contains the orfX3, orfX2, orfX1, BotR, p47, NTNH and BoNT genes. The BoNT/A4 complex is connected by dashed lines to the BoNT/bvB complex to indicate its presence within the same plasmid yet separated by about 97 kb. Partial IS elements (designated IS') are indicated in green and identified by IS family designations. Genes flanking the toxin complex include the *lyc*A, *ars*C or flagellin genes. The presence of a partial flagellin gene is designated as flagellin'. Hypothetical proteins are indicated in black. Numbers indicate the distances in nucleotides within intergenic regions.

Figure 1 illustrates the toxin cluster gene arrangements found within the strains of the four different BoNT/A1-A4 and the BoNT/B1 subtypes. The two BoNT/A1 strain gene arrangements are the same for each other, the BoNT/B1 okra strain and the published BoNT/A1 ATCC 3502 (Hall 174) genome. Within the three BoNT/A1 strains, the nucleotide sequences of the toxin cluster genes are identical for the 11.7 kb region that encodes HA70, HA17, HA33, BotR, NTNH, BoNT and their intergenic regions. In addition, comparison of the entire nucleotide sequences of BoNT/A1 Hall, ATCC 19397, and ATCC 3502 chromosomes reveals these strains exhibit 99% nucleotide identity within their entire 3.8 mb length. This high level of similarity within the toxin clusters and the genome sequences illustrates the clonal nature of these three BoNT/A1 strains. While these three BoNT/A1 strains are very highly conserved, they can be differentiated using variable number tandem repeat (VNTR) analysis [53].

**Figure 2:**
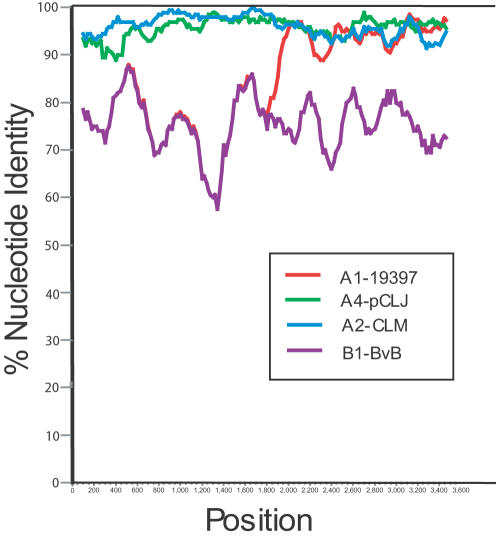
Simplot comparing the NTNH subtype sequences to the Loch Maree strain. The NTNH genes within BoNT/A1, BoNT/A2, BoNT/A4, BoNT/B1 and BoNT/bvB subtypes were compared to the NTNH gene within the BoNT/A3, Loch Maree strain. The plot shows the similarity of the NTNH genes within each of these subtypes along the length of the gene and shows a recombination event in the middle of the A1 NTNH gene (at approximately 1800 bp) where the sequence becomes more similar to the A3 NTNH in the second half of the gene.

Comparisons of the NTNH gene sequences within the BoNT/A1 and BoNT/B subtypes revealed an area of recombination in the middle of this gene. The sequence within the first half of the BoNT/B NTNH gene is similar to the NTNH sequence within the BoNT/A1 subtype; however midway or at approximately 1800 bp, an area of recombination occurs and the BoNT/B NTNH gene sequence becomes more similar to the NTNH of the BoNT/A2, BoNT/A3 or BoNT/A4 subtypes for the latter half of the gene (Figure 2 and Table 1). Such recombination events have been noted previously [54], not only with NTNH proteins from serotype A and B strains, but also within NTNH genes associated with bivalent Ab strains and strains having silent, or unexpressed, BoNT/B genes designated as A1(B) strains [12].

**Table 1:** Comparison of amino acid identities between the proteins within the toxin clusters.

Subtype	HA70	HA17	HA33	BotR	P47	NTNH		BoNT
						1^ST^ half	2^nd^ half	
**B1 vs bvB**	96%	98%	84%	100%	-----	100%	100%	96%
**bvB vs A1**	98%	98%	97%	98%	-----	99%	67%	37%
	**orfX3**	**orfX2**	**orfX1**	**BotR**	**P47**	**NTNH**		**BoNT**
						1^ST^ half	2^nd^ half	
**A1 vs A2**	-----	-----	-----	61%	-----	62%	90%	90%
**A2 vs A3**	97%	95%	97%	91%	95%	96%	93%	93%
**A3 vs A4**	95%	78%	88%	91%	75%	91%	94%	84%

Percent amino acid identities between the different BoNT subtypes are shown for each of the proteins within the toxin clusters. Amino acid identity among the A1 and B1/bvB cluster proteins (HA70, HA17, HA33, BotR, and NTNH) ranges from 83–100%. Comparison of the BoNTs in A1 versus B1 strains show only 37% amino acid identity; the NTNH comparisons for BoNT/A1 vs B1 show that the first half of the protein is similar while the second half is divergent. The amino acid identity within the toxin cluster found among the BoNT/A2-A4 subtypes ranges from 74–98%. Within the three subtypes the most similar proteins appear to be NTNH, BotR, and orfX3 (92–98%), while the orfX1, orfX2, p47, and the BoNT similarities range from 74–93%.

The three strains that represent the BoNT/A2-A4 subtypes have the same toxin complex gene arrangement that includes orfX3, orfX2, orfX1, BotR, p47, NTNH and BoNT genes (Figure 1). Pairwise comparisons of the proteins encoded by these toxin cluster genes show that there is great similarity between the BoNT/A2 and BoNT/A3 strains (Table 1). The amino acid identities between these subtypes range from 91–97%, which suggests a common origin for these two toxin clusters.

The toxin complex genes within the BoNT/A4 subtype vary in their identity to the BoNT/A2 and BoNT/A3 complex genes. With three of the BoNT/A4 cluster genes-NTNH, BotR and orfX3- an amino acid identity of 91–94% to the BoNT/A3 subtype is observed. However, within the orfX1, orfX2, p47, and BoNT only 75–88% identity is seen (Table 1). These differences in identity among the proteins within the toxin cluster of BoNT/A4 when compared to the BoNT/A2 and BoNTA3 strains indicate possible sites of genetic rearrangements or recombination events.

### Insertion Sequences

The sequence coverage allowed a thorough examination of the regions flanking the toxin complex genes to investigate possible mechanisms of genetic mobilization of the toxin cluster genes. Insertion sequence elements (IS elements) are short sequences of DNA that encode transposase enzymes that promote their translocation and contribute to genetic recombination and exchange among bacteria [55]. Table 2 lists the IS elements that were identified and Figure 1 shows their location in relation to each of the toxin gene clusters. Only partial IS elements, ranging from 23–83% homology to the full length elements, were identified within the intergenic or flanking regions of the toxin complex from each subtype. These degraded IS elements were characterized by IS family, group and closest IS homolog using the IS Finder database [52]. The location and sequences of the IS elements provided insight into the relationships among these subtypes.

**Table 2:** Insertion sequence (IS) elements identified within intergenic and flanking regions of serotype A and B strains.

Subtype	Location^1^	Intergenic Space^2^	IS Family^3^	IS Group^3^	IS Homolog^3^	Present/Full-length^4^	%Present^5^
A1	upstream	963	IS3	IS150	IS 1233	82/177	46%
	downstream	2185	IS3	IS150	ISSau2	210/521	40%
	downstream	2185	IS3	IS150	IS 1069	216/272	79%
A2	orfX1/p21	1226	IS6		ISS1Z	234/340	68%
A3	upstream	1329	IS605	IS200	IS200S	56/151	37%
	downstream	679	IS3	IS150	ISSau2	148/521	28%
	orfX1/p21	1230	IS6		ISS1Z	254/340	75%
A4	orfX1/p21	1313	IS6		ISOur1	282/340	83%
bvB	downstream	2791	IS256		ISCp1	94/383	25%
B1	downstream	2780	IS256		ISCp1	77/329	23%

1 IS element location with respect to the toxin complex.

2 Intergenic space (in nucleotides)

3 IS family, group and homolog identified using the IS Finder database.

4 The IS element showing greatest homology at the amino acid level using BLAST

5 Amino acids present/amino acids of the full-length element

IS elements present within the intergenic or flanking regions of the toxin complex from each subtype were identified and characterized by IS family and group. Only partial IS elements ranging from 23–83% homology to the full-length elements, were identified. The IS elements and their location are the same within the BoNT/A1 strains of ATCC 3502, ATCC 19397 and Hall but not shown.

In the three BoNT/A1 strains, partial IS elements were identified, one element upstream and two downstream from the toxin complex genes (Figure 1). The same location of these IS elements among the three strains shows that these A1 strains share a similar ancestor. Their degradation to 40%–79% of the full length element suggests that significant evolutionary time has occurred since their insertion into this region of the chromosome.

Although the BoNT/A1 and BoNT/B strains have similar genes that comprise their toxin clusters, the IS elements that are located upstream of the toxin complex genes differ in these two serotypes. The IS element fragments in both the BoNT/B1 and BoNT/bvB strain belong to the IS256 family and the BoNT/A1 IS element fragments are of the IS3 family, indicating independent insertion events occurred within these two serotypes.

A partial IS element between the orfX1 and the BotR gene was identified within the toxin complex genes of BoNT/A2, BoNT/A3 and BoNT/A4 subtypes (Figure 1). This partial element is in the same location and orientation within all three subtypes where 68% (BoNT/A2), 75% (BoNT/A3) or 83% (BoNT/A4) of the element remains. The similar location of this partial IS element among the A2, A3, and A4 subtypes supports a similar origin for these three subtypes. Comparisons of the nucleotide sequence within a common region of these partial IS elements of A2 versus A3, A3 versus A4, and A2 versus A4 revealed identities of approximately 98%, 90%, and 90% respectively. This suggests that the BoNT/A2 and BoNT/A3 subtypes are more closely related to each other than to the BoNT/A4 subtype.

### Plasmids

The sequence analyses for each strain revealed the presence of single plasmids within the BoNT/A3 Loch Maree strain, the BoNT/B1 okra strain, and two plasmids within the bivalent Ba4 657 strain. The circular nature of the plasmid contigs was confirmed by the presence of multiple clones containing sequence data from both the beginning and the end of the contig in the same clone. The plasmids are approximately 149 kb (pCLD within the BoNT/B1 okra strain) 267 kb (pCLK within the BoNT/A3 Loch Maree strain) and 270 kb (pCLJ within the bivalent Ba4 657 strain) and each were surprisingly found to contain the toxin complex genes. The pCLJ within the bivalent Ba4 strain contains both the BoNT/A4 and the BoNT/bvB toxin complex genes separated by approximately 97 kb; neither of these toxin complex genes was found within the chromosome. A second, smaller (10.5 kb) plasmid within the bivalent Ba4 657 strain was also identified that contains no toxin cluster genes.

**Figure 3:**
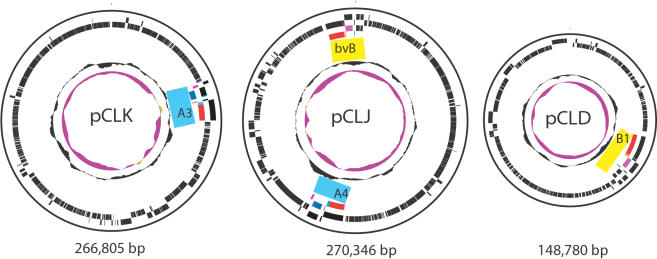
Circular plasmid maps of pCLK (Loch Maree), pCLJ (657), and pCLD (okra). These plasmid maps show the location of the neurotoxin gene complexes within each of the plasmids and the predicted sites of gene transcription. The 266,805 bp pCLK contains the BoNT/A3 gene, the 270,346 bp pCLJ contains both the BoNT/A4 and BoNT/bvB genes separated by about 97 kb and the 148,780 bp pCLD contains the BoNT/B1 gene. The data are described from the outermost circle to the innermost circle. Circles 1 and 2 show black bars to indicate predicted coding sequences on the plus strand (circle 1) and minus strand (circle 2). Circle 3 and 4 indicate the BoNT cluster genes transcribed on the plus strand and minus strand respectively. The colors used in circle 3 and 4 are the same as shown in Figure 1: NTNH and BoNT genes are red, orfX1, orfX2, orfX3 are dark blue, p47 is light blue, HA70, HA17 and HA33 are light purple and botR is orange. Circle 5 depicts the G+C content and the innermost purple circle shows the GC skew (G−C/G+C) with tan values >1 and purple<1.

Figure 3 shows a plasmid map and Table 3 describes the plasmid features for each of the larger plasmids. These three plasmids contain 191–323 predicted coding regions, of which only 23–36% have known functional properties. These plasmids do not contain rDNA genes. They contain neither the *E. coli* plasmid-like rolling-circular replication genes of *rep*A, B, C nor the *Bacillus anthracis* plasmid-like theta replication genes of *rep*X, *rep*16*5, rep*63A, *rep*E, *rep*S, and *rep*R. Although these plasmids appear to be capable of independent replication, as they have complete DNA polymerase III complex enzymes (including *dna*N) and DNA helicase II (including *pcr*A) for replication, their precise replication mechanism remains unclear.

**Table 3:** Plasmid features of pCLD, pCLJ and pCLK.

Plasmid Features	pCLD	pCLJ	pCLK
	B1 okra	Ba4	Loch Maree
Size (bp)	148,780	270,346	266,805
Coding sequence (bp)	118,215	222,681	215,214
Coding sequence (%)	79.5%	82.4%	80.7%
G+C content (%)	25.4%	25.6%	25.6%
Average orf size (bp)	619	737	666
CDSs, total	191	302	323
Number of genes with function prediction	44	108	94
Genes with function prediction (%)	23.0%	35.8% `	29.1%
Number of genes in COG	29	94	65
Genes in COG (percentage)	15.2%	31.1%	20.1%

These attributes are compared among the three plasmids and show that 80–82% of the plasmid sequence contains orfs with only 23–36% of these predicted proteins having functional properties.

**Figure 4:**
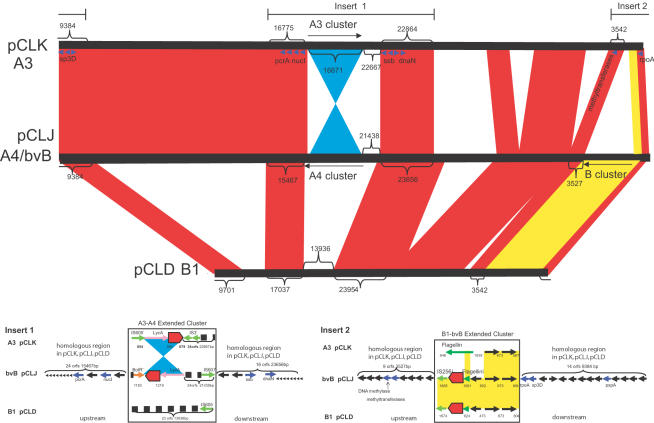
Plasmid synteny among pCLK (Loch Maree), pCLJ(657) and pCLD (okra). The upper panel illustrates regions of similarity (indicated in red) shared among these three plasmids. Regions that are not shared include the BoNT/B1 or BoNT/bvB gene clusters within the BoNT/A3 Loch Maree strain or the BoNT/A3 and BoNT/A4 gene clusters within the BoNT/B1 okra strain. These regions appear to have been deleted from pCLK and pCLD. Within pCLK and pCLJ an inversion, approximately 16.7 kb in size, has occurred where the toxin gene cluster for BoNT/A4 appears in the opposite direction from BoNT/A3. The lower panel expands the two regions designated as insert 1 and 2 in the upper panel. These regions detail the conserved plasmid sequences that flank the BoNT/A3 and BoNT/A4 extended cluster (15.4 kb upstream to 23.6 kb downstream) and the BoNT/B1 and BoNT/bvB extended cluster (3.5 kb upstream to 9.3 kb downstream).

Even though these three plasmids are of different sizes, they share significant sequence homology. The overall plasmid synteny of the three plasmids is compared in Figure 4. Sequence comparisons between the two large plasmids within the BoNT/A3 strain and the bivalent Ba4 strain illustrate the large regions within the plasmids that are very similar to each other (about 96% identity over 183 kb, which is 68% of the total length). Several of these regions flank areas where the toxin gene clusters are found. Regions that are not shared, for example where the BoNT/B1 and BoNT/bvB cluster appears within the BoNT/A3 strain or the BoNT/A3 and BoNT/A4 cluster appears within the BoNT/B1 strain, appear to have been deleted from the pCLK (BoNT/A3) or pCLD (BoNT/B1) plasmids. The BoNT/A3 and BoNT/A4 cluster in pCLJ has been replaced by an approximately 14 kb nonhomologous DNA fragment flanked by an intact IS element (IS605) in pCLD. Insertion events that may have incorporated the BoNT/A4 cluster within the bivalent plasmid and deletion events within the BoNT/B1 plasmid likely explain the differences observed in plasmid size, because the overall level of sequence identity and synteny remain high within these two plasmids.

An inversion has occurred in the region of the BoNT/A3 and BoNT/A4 plasmids that contains the toxin gene complex clusters. This inversion involves a sequence of approximately 16.7 kb, where the toxin gene cluster for BoNT/A4 appears in the opposite direction from BoNT/A3. Examination of the sequences flanking this region did not identify any palindromic or identical IS elements in the BoNT/A4 gene cluster, to indicate a possible mechanism for this event.

The identification of the BoNT/bvB and BoNT/A4 toxin complex genes within the same plasmid provides the first molecular characterization of these genes within a bivalent strain. The BoNT proteins are produced in differing amounts in bivalent strains and in the Ba4 strain the relative amount of BoNT/B produced is greater than BoNT/A4. However, it is now known that the gene copy number for the toxin complex is the same. Their location within this 270 kb plasmid separated by approximately 97 kb would indicate independent expression and this may explain the observed difference in BoNT/A4 and BoNT/bvB protein levels. In BoNT/A1 strains the NTNH and BoNT genes are positively regulated by BotR, a sigma transcription factor that immediately precedes these genes [13]. This mechanism may be the same with BoNT/bvB from bivalent strains but may not be the case with BoNT/A4, where the BotR gene is more distant from the BoNT gene and is transcribed in a different direction.

The toxin complex genes within the newly identified BoNT/A3 and BoNT/A4 subtypes were found within plasmids yet contain the same toxin complex genes as the BoNT/A2 cluster located within the chromosome. Similarly, the toxin complex arrangement within the BoNT/B1 and BoNT/bvB plasmids is the same as that within the BoNT/A1 subtypes, which is found within the chromosome. These relationships illustrate that recombination and gene transfer events have contributed to the diversity of genomic locations observed for each of these two distinct toxin complex types (A2-A3-A4, A1-B1-bvB) within these serotypes and subtypes.

### Flanking regions

The regions flanking the toxin complex genes within the plasmids and the chromosome were examined to further understand the relationships among these subtypes. The flanking regions within the plasmids contain patterns of nonhomologous regions just upstream or downstream of the toxin gene clusters, followed by areas of great homology further upstream and downstream within the plasmids (Figure 4). Comparisons of the regions containing the BoNT/A3 and BoNT/A4 gene clusters show that these two toxin gene clusters are inverted relative to each other within pCLK and pCLJ. In addition, there is a 21–23 kb region downstream of the BoNT/A3 and upstream of the BoNT/A4 clusters that shows no homology. Beyond this toxin cluster-nonhomologous region, homologous plasmid sequences flank the BoNT/A3 and BoNT/A4 gene cluster. These same sequences flank a 14 kb region within pCLD that does not include a toxin gene cluster (Figure 4).

The upstream homologous regions in the BoNT/A3 and BoNT/A4 cluster are approximately 15.5–17 kb in length, coding for 24–28 genes including the prophage maintenance system killer protein, DNA helicase II, PcrA, thermonuclease and other hypothetical proteins. The downstream regions in the BoNT/A3 and BoNT/A4 cluster are approximately 23–24 kb in length, coding for 16–20 genes including the K03111 single-strand DNA-binding protein ssb, DNA polymerase III, DnaN, cell wall-associated biofilm protein and other hypothetical proteins.

The inversion that encompasses the BoNT/A3 and BoNT/A4 complexes includes the toxin complex genes and the *lyc*A gene that is upstream of both complex clusters. The *lyc*A gene encodes autolytic lysozyme (1,4-beta-N-acetylmuramidase). This gene is also found downstream within the BoNT/A2 strain (Figure 1), but not within either the chromosome or plasmid of the BoNT/B1 strain. The similar location of the *lyc*A gene within the BoNT/A3 and BoNT/A4 strains and the overall plasmid synteny distinguish these subtypes from the BoNT/A2 subtype.

The BoNT/B1 and BoNT/bvB toxin clusters within pCLD and pCLJ are nearly identical but this toxin gene cluster is deleted in pCLK. Instead this region in pCLK contains an intact flagellin gene where only partial flagellin genes are located within both pCLD and pCLJ (Figure 4). The BoNT/B1 and BoNT/bvB cluster and nonhomologous region in pCLK is flanked upstream and downstream by homologous regions of approximately 3500 and 9400 bp, respectively (Figure 4). The upstream region of nine orfs includes DNA methylase transferase and the downstream region orfs encode for RNA polymerase alpha subunit (RpoA), stage III sporulation protein D (Sp3D), phage shock protein A (PspA) and other hypothetical proteins.

The flanking regions of the complexes of BoNT/A1, BoNT/B1 and BoNT/bvB all have similar components of a partial or intact flagellin gene, and the same three hypothetical proteins (Figure 1 and Figure 4). An intact flagellin gene can be found upstream of the BoNT complex in the three BoNT/A1 strains (Figure 1) and in the nonhomologous region of pCLK (Figure 4). Partial flagellin genes are located downstream of the BoNT/B1 and BoNT/bvB complexes. Three hypothetical proteins are upstream from the intact flagellin gene in the three BoNT/A1 strains and are the same hypothetical genes flanking the BoNT/B1 and BoNT/bvB complex. Two of these hypothetical proteins are located in the nonhomologous region within pCLK (Figure 4). The presence of these sequences near the complexes of BoNT/A1, BoNT/B1 and BoNT/bvB but distant to the BoNT/A3 and BoNT/A4 complex link these subtypes and serotypes to a similar origin and distinguish them from the BoNT/A2 strain where none of these sequences have yet been identified. Instead the chromosomally-located BoNT/A2 and plasmid-located BoNT/A3 and BoNT/A4 cluster are all flanked by the *lyc*A gene, and the BoNT/A2 cluster is further flanked upstream and downstream by intact copies of the *ars*C gene, which encodes arsenate reductase. This *ars*C gene was not identified within the flanking regions of the other strains. The location of duplicate copies of the *ars*C gene in the regions flanking the toxin complex could allow the formation of hairpin structures that mediate recombination or movement of the toxin cluster genes within the genome.

## Discussion

Sequences of eight botulinum toxin gene clusters from seven *C. botulinum* strains were analyzed to determine the arrangement of the toxin cluster genes in strains representing the BoNT/A1-A4 and BoNT/B1 subtypes, including a bivalent Ba4 strain. The genomic sequence data provided information about the arrangement of these toxin complex genes within these strains, the presence of partial IS elements within intergenic and flanking regions, and the location of these genes within the chromosome or within a plasmid.

In these seven *C. botulinum* strains there are two distinct arrangements of the toxin complex genes. The BoNT/A2, BoNT/A3 and BoNT/A4 gene clusters contain polycistronic orfX3, orfX2, orfX1, BotR, p47, NTNH, and BoNT genes. The other arrangement found within the BoNT/A1, BoNT/B1, and BoNT/bvB gene clusters contains HA70, HA17, HA33, BotR, NTNH and BoNT genes. These two very different arrangements of the toxin cluster genes are highly conserved even though they exist in very different genomic backgrounds, and illustrates the functional role each of these genes must have in the expression, protection or transport of the neurotoxin.

BoNT/A1, BoNT/B1, and BoNT/bvB complex genes show a high degree of relatedness. Amino acid identity among the nontoxin cluster proteins (HA70, HA17, HA33, BotR, and NTNH) ranges from 83–100%. However, comparisons of the BoNT protein of BoNT/A1 to BoNT/B1 strains show only 37% amino acid identity. The differences between these BoNT genes and their associated toxin complex genes indicate that recombination events have contributed to the diversity between these serotypes. Examination of NTNH sequences from various BoNT/A and BoNT/B strains has identified a recombination ‘hot spot” occurring midway in the gene [3]. In these strains a recombination event was also identified midway within the BoNT/A1 NTNH gene resulting in a high degree of similarity to the BoNT/B1 and BoNT/bvB NTNH gene within the first half of the gene and greater similarity to the BoNT/A2, BoNT/A3 or BoNT/A4 NTNH in the latter half of the gene.

The similarity within the arrangements of the toxin complex genes, their amino acid sequences and the presence of similar proteins and partial IS elements in their flanking regions suggest that the BoNT/A1, BoNT/B1, and BoNT/bvB clusters are from a common lineage but mutation and/or recombination events have contributed to their evolution as distinct serotypes and subtypes.

The other conserved gene cluster arrangement is found among the BoNT/A2-A4 subtypes. In these subtypes, amino acid identity within the cluster genes ranges from 74–98%. Within the three subtypes the most similar proteins appear to be NTNH, BotR, and orfX3 (92–98%), while the orfX1, orfX2, p47, and the BoNT similarities range from 74–93%. The similar gene arrangement and location of a partial IS element belonging to the IS6 family, between BotR and orfX1 in these three subtypes also suggests a common ancestry. This partial IS element and the amino acid identities within the toxin complex proteins show that the BoNT/A2 and BoNT/A3 gene clusters are more similar to each other than to the BoNT/A4.

These BoNT/A2-A4 subtypes exhibit a complex history of recombination. Recombination events include an inversion of the entire BoNT/A3 and BoNT/A4 complex within a plasmid background, relocation of the *lyc*A gene in the chromosomal flanking region of the BoNT/A2 subtype, the presence of different flanking regions containing different partial IS elements, and multiple gene duplication events involving the *ars*C gene that resides both upstream and downstream of the BoNT/A2 cluster.

The presence of partial IS elements in flanking regions and/or within the toxin gene clusters suggests that these mobile elements may have had a role in the transfer of these genes during the evolution of clostridial species [15], [56]. IS elements, which can contribute to gene mobility, flank the toxin gene clusters within the BoNT/A1 and BoNT/A3 strains, and are found downstream of the clusters in the BoNT/B strains. Their degradation to 23–83% of their original length suggests that their insertion occurred early during the evolution of *C. botulinum*. Since this time, paired IS elements could have been lost and host genetic preferences (G+C content, di-nucleotide frequency, codon bias) could have been acquired which might mask evidence of earlier gene transfer events [57].

The presence of the toxin cluster genes within plasmids appears just as common as their presence within the chromosome. In these serotype A and B strains the BoNT/A1-B1-bvB complex can be found within the plasmid or the chromosome. Similarly the BoNT/A2-A3-A4 complex can be found in both locations. There is no clear evidence to support whether the genes within these toxin complexes originated within the chromosome or plasmid, however the discovery of BoNT-encoding plasmids provides insight into understanding the amount of apparent recombination and movement of these genes among clostridial species. Earlier reports of a chromosomal location for the toxin cluster genes from serotypes A, B, E, and F were based on experiments using techniques for the isolation of relatively small plasmids from genomic DNA preparations [58]. Using pulsed-field gel electrophoresis and Southern hybridization techniques the presence of the BoNT/A3, BoNT/A4 and BoNT/bvB genes within plasmids has recently been confirmed [59]. Here the presence of plasmids was discovered after genomic sequencing of these strains. These findings suggest that other plasmids may exist within *C. botulinum* strains.

Sequence comparisons show that these plasmids appear to be unique. They have no similarity to the toxin-encoding plasmids within *C. tetani* [56], *C. perfringens* [60], or the BoNT/C and BoNT/D-encoding phage within *C. botulinum* [61]. Other neurotoxin-encoding plasmids identified within *C. botulinum* are found within the serotype G strains [62]. Since none of these BoNT/G containing plasmids have yet been sequenced it is unknown whether these share any similarity to pCLD, pCLJ or pCLK. Although the exact mechanism of their replication is not understood, these large plasmids appear to be in low copy number based on the relative sequence coverage of the plasmid and chromosome. Their presence and/or expression of BoNT must confer an advantage to the host to have been maintained in these strains.

Whether within a plasmid or chromosome, all of the strains (except the BoNT/A2 strain) share flanking regions encoding two or three hypothetical proteins and an intact or partial flagellin gene. This relationship suggests a common ancestor that may have contained these sequences within a plasmid, phage or chromosome. In pCLK an intact flagellin gene and two of the three hypothetical proteins are located in a region distant to the BoNT/A3. This region contains homologous plasmid sequences both upstream and downstream and suggests a toxin gene could have been deleted in this region of the plasmid that contains the BoNT/B1 or BoNT/bvB cluster in pCLJ or pCLD.

The identification of both the BoNT/bvB and BoNT/A4 toxin gene clusters within the same plasmid in the Ba4 strain provides an example of two distinct toxin clusters located within one bivalent strain and provides insight into the possible nature of other bivalent strains. Their presence within the same plasmid having the same copy number within the cell cannot explain the different levels of BoNT/bvB and BoNT/A4 produced in relation to each other. The two gene clusters are distant from each other and likely controlled via different regulatory systems, resulting in differing toxin expression levels.

These analyses of genomic sequence data elucidate some of the mechanisms active within the *C. botulinum* family that have resulted in a surprising range of genetic diversity within the few proteins that make up the BoNT cluster. Genomic sequencing has identified the arrangement of the toxin complex genes in these strains, characterized their intergenic and flanking sequences, identified recombination events and revealed the presence of neurotoxin-containing plasmids in three of these strains. With the exception of BoNT/A4, the neurotoxins produced in these strains show equivalent toxicity regardless of toxin gene, or gene cluster arrangement or location. The gene cluster arrangements appear to have successfully evolved to allow the survival and expression of different BoNTs within these organisms. Their movement into different genetic backgrounds including different species may be mediated by the presence of IS elements, duplicated genes flanking the toxin complex, and the presence of the toxin genes within plasmids. This has resulted in a surprising amount of diversity within the many bacterial strains that comprise *C. botulinum*.
